# Awareness of Stroke Signs and Symptoms and Calling 9-1-1 Among US Adults: National Health Interview Survey, 2009 and 2014

**DOI:** 10.5888/pcd16.180564

**Published:** 2019-06-20

**Authors:** Ashruta Patel, Jing Fang, Cathleen Gillespie, Erika Odom, Sallyann Coleman King, Cecily Luncheon, Carma Ayala

**Affiliations:** 1 Division for Heart Disease and Stroke Prevention, National Center for Chronic Disease Prevention and Health Promotion, Centers for Disease Control and Prevention, Atlanta, Georgia; 2 IHRC, Inc, Atlanta, Georgia

## Abstract

**Introduction:**

Early recognition of stroke symptoms and recognizing the importance of calling 9-1-1 improves the timeliness of appropriate emergency care, resulting in improved health outcomes. The objective of this study was to assess changes in awareness of stroke symptoms and calling 9-1-1 from 2009 to 2014.

**Methods:**

We analyzed data among 27,211 adults from 2009 and 35,862 adults from 2014 using the National Health Interview Survey (NHIS). The NHIS included 5 questions in both 2009 and 2014 about stroke signs and symptoms and one about the first action to take when someone is having a stroke. We estimated the prevalence of awareness of each symptom, all 5 symptoms, the importance of calling 9-1-1, and knowledge of all 5 symptoms plus the importance of calling 9-1-1 (indicating recommended stroke knowledge). We assessed changes from 2009 to 2014 in the prevalence of awareness. Data analyses were conducted in 2016.

**Results:**

In 2014, awareness of stroke symptoms ranged from 76.1% (sudden severe headache) to 93.7% (numbness of face, arm, leg, side); 68.3% of respondents recognized all 5 symptoms, and 66.2% were aware of all recommended stroke knowledge. After adjusting for sex, age, educational attainment, and race/ethnicity, logistic regression results showed a significant absolute increase of 14.7 percentage points in recommended stroke knowledge from 2009 (51.5%) to 2014 (66.2%). Among US adults, recommended stroke knowledge increased from 2009 to 2014.

**Conclusion:**

Stroke awareness among US adults has improved but remains suboptimal.

**Summary**

**What is already known on this topic?**

Early recognition of stroke symptoms and the importance of calling 9-1-1 improves cardiovascular outcomes. In 2014, US age-adjusted awareness rate of stroke symptoms and knowing to call 911 was 66% and was higher among females, whites, and individuals with health insurance.

**What is added by this report?**

Awareness of all stroke symptoms and knowledge of calling 9-1-1 among US adults increased by 14.7 percentage points from 2009 to 2014, and the increase was observed in almost all subgroups.

**What are the implications for public health practice?**

In the United States, the awareness of stroke improved over the past several years. Educational activities to sustain the high levels of awareness of stroke should be continued.

## Introduction

Stroke is the fifth leading cause of death in the United States. According to the American Heart Association, stroke kills nearly 140,000 people each year, accounts for 1 of every 20 deaths, and is the leading cause of long-term disability (1). Public awareness of the symptoms of stroke and how to access emergency assistance is essential to increase the likelihood of achieving a favorable outcome. Several studies of public health campaigns for stroke awareness, including distribution of booklets and animations about early symptoms of stroke, have been conducted to improve population knowledge and decrease the likelihood of prehospital delays (2,3).

The US Department of Health and Human Service’s Healthy People 2020 (HP2020) includes heart disease and stroke objectives: 1) increase the prevalence of awareness of 5 early warning symptoms of a stroke, 2) increase the prevalence of awareness of the importance of accessing rapid emergency care by calling 9-1-1, and 3) increase the prevalence of awareness of both the 5 early warning symptoms of a stroke and the importance of accessing rapid emergency care by calling 9-1-1.

The prevalence by population of awareness of the signs of stroke has been reported (4). We used National Health Interview Survey (NHIS) data to assess the change in awareness of stroke signs and symptoms from 2009 to 2014. Our objectives were to 1) examine changes in prevalence of awareness of stroke signs and symptoms from 2009 to 2014, 2) describe changes in prevalence of the HP2020 stroke awareness measures, and 3) examine sociodemographic and health-related factors associated with changes in prevalence of the HP2020 stroke awareness measures from 2009 to 2014.

## Methods

We used publicly available NHIS data. All data were de-identified, so institutional board review approval was not needed (5). The NHIS is an annual survey that uses a multistage probability sampling design and that collects health-related data on the US civilian noninstitutionalized population (6). 

The 2009 and 2014 NHIS included questions about stroke symptoms and the best action to take when someone may be having a stroke. Participants were asked which of the following were symptoms that someone may be having a stroke: 1) sudden numbness or weakness of face, arm, leg, especially on one side of the body; 2) sudden confusion or trouble speaking; 3) sudden trouble seeing in one or both eyes; 4) sudden trouble walking, dizziness, or loss of balance; and 5) sudden severe headache with no known cause.

Respondents who answered yes to these questions were categorized as knowing the symptoms of a stroke. Those who answer no or “don’t know” were categorized as not knowing the symptoms. Participants were then asked, “If you thought someone was having a stroke, what is the best thing to do right away?” Respondents who answered “Call 9-1-1 (or other emergency number)” were classified as knowing the importance of calling 9-1-1; participants who answered “advise them to drive to the hospital,” “advise them to call their physician,” or “call spouse or family member” were classified as not knowing the importance (6). Respondents who knew all 5 stroke symptoms and who knew the importance of calling 9-1-1were categorized as having “recommended stroke knowledge.”

Demographic characteristics were sex (male, female); age in years (18–44, 45–64, ≥65); race/ethnicity (non-Hispanic white, non-Hispanic black, non-Hispanic Asian, Hispanic, other); completed education among adults aged 25 years or older (less than high school, high school graduate, some college, college graduate); family income-to-poverty ratio (calculated by dividing the family income by the US Census Bureau poverty threshold and categorized as <1.0, 1.0 to <2.0, or ≥2.0); and marital status (married or living with a partner, not married or living with a partner). Participants younger than 25 were included in the sample but not analyzed as a subgroup. Geographic region was defined based on US Census Bureau classifications: Northeast (Connecticut, Maine, Massachusetts, New Hampshire, New Jersey, New York, and Pennsylvania, Rhode Island, Vermont), Midwest (Illinois, Indiana, Iowa, Kansas, Michigan, Minnesota, Missouri, Nebraska, North Dakota, Ohio, South Dakota, and Wisconsin), South (Delaware, District of Columbia, Florida, Georgia, Maryland, North Carolina, South Carolina, Virginia, West Virginia, Alabama, Kentucky, Mississippi, Tennessee, Arkansas, Louisiana, Oklahoma, and, Texas), and West (Alaska, Arizona, California, Colorado, Hawaii Idaho, Montana, Nevada, New Mexico, Oregon, Utah, Washington, and Wyoming) (7). 

Measures of access to health care included having a usual place to go for health care (yes, no); having health insurance (yes, no); and deferred medical care in the past 12 months due to cost (yes, no). Measures of health status included self-reported health status (good to excellent, fair to poor) and self-reported history of major cardiovascular disease (yes, no).

We estimated the prevalence of awareness of the stroke symptoms and the prevalence of the HP2020 objectives: knowledge of all 5 stroke symptoms, the importance of calling 9-1-1, and recommended stroke knowledge. We used univariate, Satterthwaite-adjusted *χ* ^2^ tests to assess crude (unadjusted) differences in demographic characteristics from 2009 to 2014. We used logistic regression models, adjusted for demographic characteristics (sex, age, race/ethnicity, and educational attainment) to estimate the change in prevalence from 2009 to 2014 and the corresponding adjusted prevalence ratios of recommended stroke knowledge by the selected characteristics. 

All analyses were conducted following guidelines from the National Center for Health Statistics (6). Analyses were weighted to be representative of the US noninstitutionalized civilian population, and we used SAS (version 9.3) and SAS-callable SUDAAN (version 11.0, Research Triangle Institute) to account for the complex survey design (8). Analyses were conducted using *t* test for difference in prevalence from 2009 to 2014 and adjusted for sex, age, race/ethnicity, and education. Our analysis included 27,211 adults for 2009 and 35,862 adults for 2014. Data analyses were conducted in 2016. All statistical tests were 2-tailed, and we defined significance at *P* < .05.

## Results

More respondents in 2014 than in 2009 were aged 65 years or older, were Hispanic or non-Hispanic Asian, were college graduates, had a family income-to-poverty ratio of less than 1.0, and were not married or living with a partner (Table 1). Respondents with the largest change in recommended stroke knowledge from 2009 to 2014 were female, were racial/ethnic minorities, had lower educational attainment, had lower income, reported worse health status, had limited access to health care, had no health insurance, deferred medical care due to cost, lived in the Northeast, and had a history of major cardiovascular disease (Table 2). 

**Table 1:** Characteristics^a^ of Respondents, Awareness of Stroke Signs and Symptoms and Calling 9-1-1, National Health Interview Survey, 2009 and 2014

Characteristic	2009 (N = 27,211)	2014 (N = 35,862)	*P* Value^b^
	No. (%) [Standard Error]		
**Sex**			
Male	12,020 (48.2) [0.4]	16,010 (48.2) [0.4]	.87
Female	15,191 (51.8) [0.4]	19,852 (51.8) [0.4]	
**Age, y**			
18–44	12,553 (48.5) [0.5]	15,384 (46.9) [0.5]	<.001
45–64	9,286 (34.9) [0.4]	12,034 (34.4) [0.4]	
≥65	5,372 (16.6) [0.3]	8,444 (18.7) [0.3]	
**Race/ethnicity^c^**			
Non-Hispanic white	16,041 (69.2) [0.5]	22,647 (66.5) [0.5]	<.001
Non-Hispanic black	4,348 (11.7) [0.3]	4,893 (11.9) [0.3]	
Non-Hispanic Asian	927 (2.5) [0.1]	1,175 (3.2) [0.2]	
Hispanic	5,055 (13.6) [0.3]	5,920 (15.2) [0.3]	
Other	840 (2.8) [0.2]	1,227 (3.2) [0.1]	
**Completed education^d^**			
Less than high school	4,165 (12.5) [0.3]	4,985 (11.6) [0.3]	<.001
High school graduate	6,560 (24.0) [0.4]	8,354 (22.0) [0.3]	
Some college	6,977 (25.1) [0.3]	9,500 (25.1) [0.3]	
College graduate	6,743 (25.6) [0.4]	9,752 (28.6) [0.4]	
<25 years	2,766 (12.7) [0.4]	3,271 (12.6) [0.3]	
**Family income-to-poverty ratio^e^**			
<1.0	4,241 (11.6) [0.3]	5,990 (13.0) [0.3]	<.001
1.0 to <2.0	4,644 (15.3) [0.3]	7,160 (17.8) [0.3]	
≥2.0	16,007 (64.8) [0.5]	20,932 (63.9) [0.5]	
Missing	2,319 (8.2) [0.2]	1,780 (5.3) [0.2]	
**Marital status**			
Married or living with partner	13,840 (61.6) [0.4]	17,952 (60.5) [0.4]	.03
Not married or living with partner	13,371 (38.4) [0.4]	17,910 (39.5) [0.4]	
**Health status**			
Good to excellent	23,203 (87.1) [0.3]	30,643 (87.5) [0.2]	.16
Fair to poor	4,008 (12.9) [0.3]	5,219 (12.5) [0.2]	
**Have a usual place to obtain health care**			
Yes	22,838 (83.9) [0.3]	30,935 (86.4) [0.3]	<.001
No	4,373 (16.1) [0.3]	4,927 (13.6) [0.3]	
**Health insurance**			
Yes	22,209 (82.3) [0.3]	31,009 (86.6) [0.3]	<.001
No	5,002 (17.7) [0.3]	4,853 (13.4) [0.3]	
**Medical care deferred due to cost^f^**			
Yes	3,787 (12.6) [0.3]	3,854 (9.4) [0.2]	<.001
No	23,424 (87.4) [0.3]	32,008 (90.6) [0.2]	
**Region**			
Northeast	4,500 (17.5) [0.5]	5,788 (17.3) [0.4]	.09
Midwest	6,154 (24.3) [0.5]	7,645 (23.0) [0.5]	
South	9,973 (35.9) [0.7]	12,637 (37.3) [0.5]	
West	6,584 (22.4) [0.5]	9,792 (22.4) [0.4]	
**History of major cardiovascular disease^g^**			
Yes	3,806 (13.3) [0.3]	5,238 (12.9) [0.2]	.23
No	23,405 (86.7) [0.3]	30,624 (87.1) [0.2]	

a Analytic sample includes adults aged ≥18 years.

b *P* values for characteristics and year determined by using univariate Satterthwaite-adjusted χ^2^ test of independence.

c Non-Hispanic Asian includes Chinese, Korean, Vietnamese, Japanese, and other Asian subgroups. “Other” race/ethnicity includes American Indian, Alaska Native, Asian Indian, Pacific Islander, other race, and multiple races.

d Completed education assessed for adults ≥25 years of age; participants younger than 25 were included in the sample but not analyzed as a subgroup. Participants with unknown education level were excluded.

e Family income-to-poverty ratio is the ratio of the family’s income to the appropriate federal poverty threshold.

f Medical care deferred due to cost was assessed with the question “During the past 12 months, has medical care been delayed because of worry about the cost?”

g History of major cardiovascular disease was assessed with the question, “Have you ever been told by a doctor or other health professional that you had coronary heart disease, angina pectoris, myocardial infarction, any kind of heart condition or heart disease, or stroke?”

**Table 2:** Logistic Regression Analysis of Recommended Stroke Knowledge^a^ Between 2009 and 2014, National Health Interview Survey

Characteristic	Recommended Stroke Knowledge	
	Percentage-Point Change^b^ (Standard Error)	Adjusted Prevalence Ratio^b^ (95% Confidence Interval)
**Total**	14.7 (0.6)	1.29 (1.26–1.32)
**Sex **		
Male	14.7 (0.9)	1.30 (1.26–1.34)
Female	14.9 (0.8)	1.28 (1.24–1.31)
**Age, y **		
18–44	15.1 (0.9)	1.31 (1.27–1.35)
45–64	14.5 (0.9)	1.26 (1.23–1.30)
≥65	14.4 (1.3)	1.28 (1.23–1.34)
**Race/ethnicity^c^**		
Non-Hispanic white	13.9 (0.8)	1.25 (1.22–1.28)
Non-Hispanic black	15.3 (1.5)	1.32 (1.25–1.40)
Non-Hispanic Asian	22.0 (2.9)	1.66 (1.44–1.90)
Hispanic	17.2 (1.4)	1.46 (1.37–1.56)
Other	16.4 (3.0)	1.38 (1.22–1.57)
**Completed education^d^**		
Less than high school	18.3 (1.5)	1.50 (1.40–1.60)
High school graduate	14.6 (1.1)	1.30 (1.25–1.35)
Some college	15.0 (1.0)	1.27 (1.23–1.32)
College graduate	12.4 (1.0)	1.21 (1.17–1.25)
**Family income-to-poverty ratio^e^**		
<1.0	18.6 (1.4)	1.46 (1.37–1.55)
1.0 to <2.0	17.1 (1.4)	1.38 (1.31–1.46)
≥2.0	13.4 (0.8)	1.24 (1.21–1.27)
**Marital status **		
Married or living with partner	13.9 (0.8)	1.26 (1.23–1.30)
Not married or living with partner	16.1 (0.9)	1.33 (1.29–1.37)
**Health status**		
Good to excellent	14.7 (0.7)	1.28 (1.25–1.31)
Fair to poor	15.5 (1.5)	1.34 (1.27–1.42)
**Have a usual place to obtain health care**		
Yes	14.3 (0.7)	1.27 (1.24–1.30)
No	16.9 (1.5)	1.39 (1.30–1.47)
**Health insurance**		
Yes	14.4 (0.7)	1.27 (1.24–1.30)
No	16.2 (1.4)	1.37 (1.29–1.45)
**Medical care deferred due to cost^f^**		
Yes	16.6 (1.5)	1.33 (1.26–1.40)
No	14.5 (0.7)	1.28 (1.25–1.31)
**Region **		
Northeast	19.2 (1.5)	1.40 (1.32–1.48)
Midwest	11.1 (1.1)	1.20 (1.16–1.25)
South	15.3 (1.1)	1.29 (1.24–1.33)
West	14.0 (1.4)	1.30 (1.23–1.37)
**History of major cardiovascular disease^g ^**		
Yes	16.6 (1.4)	1.33 (1.26–1.39)
No	14.5 (0.7)	1.28 (1.25–1.31)

a Based on Healthy People 2020 Heart Disease and Stroke Objective number 17.1: recommended stroke knowledge.

b Percentage-point change reflects the difference in prevalence between 2014 and 2009. Adjusted prevalence ratios reflect the prevalence ratio of awareness in 2014 compared with 2009.

c Non-Hispanic Asian includes Chinese, Korean, Vietnamese, Japanese, and other Asian subgroups. “Other” race/ethnicity includes American Indian, Alaska Native, Asian Indian, Pacific Islander, other race, and multiple races.

d Completed education assessed for adults ≥25 years of age; participants younger than 25 were included in the sample but not analyzed as a subgroup. Participants with unknown education level were excluded.

e Family income-to-poverty ratio is the ratio of the family’s income to the appropriate federal poverty threshold.

f Medical care deferred due to cost was assessed with the question “During the past 12 months, has medical care been delayed because of worry about the cost?”

g History of major cardiovascular disease was assessed with the question, “Have you ever been told by a doctor or other health professional that you had coronary heart disease, angina pectoris, heart attack (MI), any kind of heart condition or heart disease, or stroke?”

Among the subgroups examined with statistically stable estimates in the change in prevalence, the adjusted prevalence increase was smallest among college graduates (12.4 percentage points) and largest (18.6 percentage points) among those with a family income-to-poverty ratio of less than 1.0. Non-Hispanic Asians had an adjusted prevalence increase of 22.0 percentage points. Adults in 2014 were 29% more likely to be aware of all recommended stroke knowledge, compared with 2009. The increase in the adjusted prevalence ratios of recommended stroke knowledge in 2014 compared with 2009 were significant across all sociodemographic subgroups. The adjusted prevalence ratios ranged from 1.20 among residents of the Midwest to 1.66 among non-Hispanic Asians. A similar trend was seen among changes in all 5 symptoms and calling 9-1-1.

**Figure 1:**
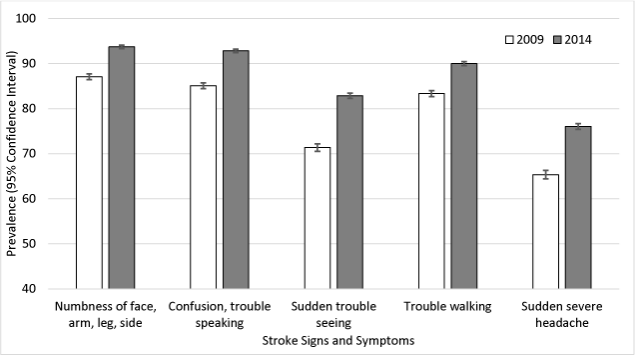
Prevalence of stroke symptom awareness, National Health Interview Survey, 2009 and 2014. Stroke symptom awareness was assessed with the question, “Which of the following would you say are the symptoms that someone may be having a stroke?” Response options were numbness of face, arm, leg, or side; confusion or trouble speaking; sudden trouble seeing; trouble walking; and sudden severe headache. Analyses were conducted using *t* test for difference in prevalence from 2009 to 2014 and adjusted for sex, age, race/ethnicity, and education.

Awareness of all 5 stroke symptoms improved from 2009 to 2014 (Figure 1). The most commonly recognized symptom in both years was numbness of face, arm, leg, or side. The least recognized symptom was sudden, severe headache. The largest increase in symptom awareness was observed for sudden trouble seeing, which increased by 11.5 percentage points.

**Table:** 

Response	2009	2014	*P* Value
	% (95% Confidence Interval)		
Numbness of face, arm, leg, or side	87.1 (86.4–87.7)	93.7 (93.3–94.1)	<.001
Confusion or trouble speaking	85.1 (84.5–85.8)	92.8 (92.4–93.2)	<.001
Sudden trouble seeing	71.4 (70.5–72.2)	82.9 (82.3–83.4)	<.001
Trouble walking	83.4 (82.7–84.0)	90.0 (89.6–90.5)	<.001
Sudden severe headache	65.4 (64.5–66.3)	76.1 (75.4–76.7)	<.001

**Figure 2:**
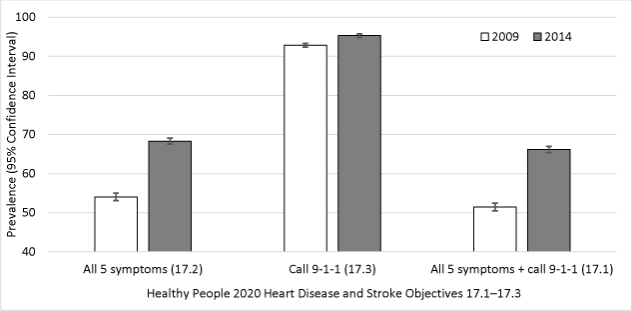
Prevalence of knowledge of Healthy People 2020 heart disease and stroke objectives 17.1–17.3, National Health Interview Survey, 2009 and 2014. Knowledge of all 5 stroke symptoms (ie, numbness of face, arm, leg, or side; confusion or trouble speaking; sudden trouble seeing; trouble walking; and sudden severe headache) was assessed with the question, “Which of the following would you say are the symptoms that someone may be having a stroke?” Awareness of the importance of calling 911 was assessed with the question, “If you thought someone was having a stroke, what is the best thing to do right away?” Participants were defined as aware if they answered, “Call 911 or other emergency number.” Recommended stroke knowledge was defined as correct identification of all 5 stroke symptoms and knowing the importance of calling 911 or other emergency number. Analyses were conducted using *t* test for difference in prevalence from 2009 to 2014 and adjusted for sex, age, race/ethnicity, and education.

The prevalence of recognizing all 5 stroke symptoms increased by 14.2 percentage points from 2009 to 2014 (Figure 2). The prevalence of calling 9-1-1 if someone was having a stroke increased by 2.5 percentage points from 2009 to 2014. The prevalence of recommended stroke knowledge increased by 14.7 percentage points from 2009 to 2014.

**Table:** 

Response	2009	2014	*P* Value
	% (95% Confidence Interval)		
All 5 symptoms	54.1 (53.1–55.0)	68.3 (67.5–69.1)	<.001
Call 9-1-1	92.8 (92.3–93.3)	95.3 (94.8–95.7)	<.001
All 5 symptoms + call 9-1-1	51.5 (50.5–52.5)	66.2 (65.4–67.0)	<.001

## Discussion

Our findings showed that awareness of all 5 stroke signs and symptoms and the importance of calling 9-1-1 improved significantly from 2009 to 2014. The HP2020 targets for improvement in the 3 objectives examined were met and exceeded. Our results for HP2020 goal 17.1 showed a prevalence of 66.2% in 2014, exceeding the HP2020 goal of 56.4%. We observed a prevalence of 68.3% in 2014 and an adjusted improvement of 14.3 percentage points for HP2020 goal 17.2, exceeding the HP2020 goal. Our findings for HP2020 goal 17.3 showed a prevalence of 95.3% in 2014, which exceeded the HP2020 goal by 0.6% and an adjusted improvement of 2.5 percentage points over baseline. In addition, there was an adjusted improvement of 14.7 percentage points in recommended stroke knowledge. 

We saw significant increases in awareness of recommended stroke knowledge among most groups. The largest improvements were among those aged 18 to 44 years, non-Hispanic Asians and Hispanics, those with less than high school education, those who have a family income-to-poverty ratio of less than 1.0, those who are not married or living with a partner, individuals who do not have access to health care, those who deferred medical care due to cost, and those who have a history of cardiovascular disease. Groups demonstrating the least amount of change were non-Hispanic whites, college graduates, those with a family income-to-poverty ratio of 2.0 or higher, and those residing in the Midwest.

Integrative review studies analyzing data from 1966 to 2008 also found an increased prevalence of individuals who would contact emergency medical services at the onset of stroke symptoms, despite lower levels of knowledge of recognizing and preventing stroke in older people, people from racial/ethnic minority groups, and people with lower levels of education (9). The Paul Coverdell National Acute Stroke Program aims to develop high-quality, statewide stroke systems of care and promotes public awareness of stroke symptoms and the importance of calling 9-1-1 (10). Another set of symptoms of stroke is contained in the F.A.S.T. (ie, Face, Arms, Speech, Time) mnemonic, which helps remind the public about certain signs seen in stroke patients. The American Stroke Association continues to promote the importance of stroke awareness, calling 9-1-1, and stroke education (11). Multiple factors have helped promote awareness of stroke symptoms and could have contributed to the increase in awareness observed from 2009 to 2014 (9–11). 

Several studies have analyzed stroke awareness and the importance of public health campaigns. Gao et al suggested the necessity to emphasize individual stroke symptoms in stroke awareness campaigns when targeting populations classified by risk (12). Community-level behavioral interventions that include motivational exercises related to calling 9-1-1 in the presence of stroke symptoms also could be beneficial (13). Although most individuals report knowing the signs and symptoms of stroke and the importance of calling 9–1-1, large-scale and small-scale studies show that less than 60% of hospitalized stroke patients are transported to the emergency department by emergency medical services (14,15). 

Although an individual may be aware of one sign or symptom, the prevalence of knowing all 5 was still low, even though HP2020 goals were met. Knowing all 5 signs and symptoms as opposed to one may be important in follow-through to call 9-1-1. We need continued efforts in education on knowing all 5 signs and symptoms, in addition to calling 9-1-1. Furthermore, more than 90% of individuals knew to call 9-1-1 if someone was having a stroke; however, if they are unaware that some of the symptoms were related to stroke, they may not do so.

This paradox in knowledge of signs and symptoms, calling 9-1-1, and follow-through to arrival by emergency medical services was more likely to occur among racial/ethnic minorities and women, and it reduces opportunities for access to life-saving treatment and increases chances for morbidities (14,15). As a result, stroke education campaigns that include messages on stroke signs/symptoms, calling 9-1-1, as well as the time-sensitive response needed for stroke victims to access life-saving treatment may be warranted. 

Our study has limitations. NHIS uses closed-ended questions, which limits the amount of information collected, and results are also subject to recall bias. The NHIS sample comprises noninstitutionalized adults, so respondents who are not living in the community or who have cognitive or physical limitations may be not be represented. A strength of our study is the use of a large, nationally representative sample, which provided statistical power. 

Our study demonstrated that public knowledge of stroke awareness improved over the past several years, and results indicate a need to continue educational activities to sustain the high levels of recommended stroke knowledge among the general population. Future campaigns could develop methods focus on symptoms that the public has less stroke knowledge, such as a sudden severe headache, about to help individuals learn about the signs associated with stroke creating a better understanding of stroke among the general population (16).
